# Supplementary material for: Pollen-food allergy syndrome and component sensitization in adolescents: A Japanese population-based study
Source: PLoS One. 2021 Apr 14;16(4):e0249649. doi: 10.1371/journal.pone.0249649 (PMC8046202; doi:10.1371/journal.pone.0249649)
Supplement: Attachment — Submitted filename: 1st Review. 1.12.21.docx [file pone.0249649.s001.docx]

1^st^ Review

Summary:

Kiguchi et al conducted a cross-sectional study using questionnaires on pollen allergy, PFAS, and OAS as well as ImmunoCAP SAC to assess IgE component sensitization among 13-year-old adolescents residing in the Tokyo region. Pollen-food allergy syndrome (PFAS) is termed for oral allergy syndrome that occurs among those with pollen allergy. The authors reported a high prevalence of PFAS than previously thought. Kiwi and pineapple were found to be common causal foods. The authors discussed the possible cross reactivity between cedar allergy with common PFAS causative foods. Despite the high PFAS prevalence, the understanding of PFAS among the participants appear to be low. The authors call for the development of prevention strategies and the education of the public regarding PFAS.

Major concerns:

1. The authors mention that their findings suggest “PFAS is becoming more common in adolescents than previously thought”. However, the paper does not discuss statistical evidence that suggest a change in prevalence. I believe it would be helpful for the reader if the paper discussed relevant statistics that shows a change in prevalence over time.
2. Background information regarding IgE component sensitization would be helpful for those who are unfamiliar to the field of immunology.

Minor concerns:

1. There appears to be some run-on sentences.
2. The current format of Table 9 makes the words and numbers fall into two rows. Will this table be published longitudinally to allow for the words and numbers to fit into one row for easy reading?
